# Supine Hypertension as a Predictor of Atrial Fibrillation: A UK Biobank Cohort Study

**DOI:** 10.1016/j.jacadv.2026.102960

**Published:** 2026-07-09

**Authors:** Kyung-Yeon Lee, Tae-Min Rhee, JungMin Choi, Hyo-Jeong Ahn, So-Ryoung Lee, Heesun Lee, Su-Yeon Choi, Seil Oh, Gregory Y.H. Lip, Eue-Keun Choi

**Affiliations:** a Department of Internal Medicine, Korea University Guro Hospital, Seoul, Republic of Korea; b Department of Internal Medicine, Seoul National University College of Medicine, Seoul, Republic of Korea; c Department of Internal Medicine, Seoul National University Hospital, Seoul, Republic of Korea; d Department of Internal Medicine, Seoul National University Hospital Healthcare System Gangnam Center, Seoul, Republic of Korea; e Liverpool Centre for Cardiovascular Science at University of Liverpool, Liverpool John Moores University and Liverpool Chest and Heart Hospital, Liverpool, United Kingdom; f Department of Clinical Medicine, Aalborg University, Aalborg, Denmark

**Keywords:** arrhythmia, atrial fibrillation, cardiovascular disease, hypertension, supine hypertension

## Abstract

**Background:**

Atrial fibrillation (AF) is a common arrhythmia strongly associated with blood pressure (BP), yet most current assessments focus only on seated BP measurements. Supine BP may offer additional predictive value by capturing positional or autonomic variations often missed in standard evaluations.

**Objectives:**

This study aimed to investigate the association between supine BP and incident AF.

**Methods:**

We analyzed 24,901 UK Biobank participants without prior cardiovascular disease or antihypertensive treatment, categorizing them into 4 groups based on the presence of seated and/or supine hypertension: normal BP, seated-only hypertension, supine-only hypertension, and both seated and supine hypertension. The primary endpoint was incident AF over a median follow-up of 4.5 years.

**Results:**

Among 24,901 participants (mean age 63.1 ± 7.7 years; 44.0% male), supine systolic BP was independently associated with incident AF (adjusted HR [aHR]: 1.12 per 10 mm Hg increase; 95% CI: 1.05-1.20), whereas seated systolic BP showed a weaker association. Supine hypertension (aHR: 1.38; 95% CI: 1.09-1.75) and both seated and supine hypertension (aHR: 1.35; 95% CI: 1.01-1.81) significantly increased AF risk, whereas seated hypertension alone did not. Supine hypertension improved AF risk prediction, increasing Harrell C-index (0.553 vs 0.576; *P* = 0.033) and net reclassification improvement (22.3%; *P* < 0.001). In participants <65 years, supine hypertension showed the strongest association with AF, but no significant association was observed in older adults.

**Conclusions:**

Supine hypertension is independently associated with increased AF risk, especially in younger adults. Seated hypertension was not independently associated with AF risk after adjustment. These findings suggest that supine BP may capture prognostic information beyond seated BP measurements.

Atrial fibrillation (AF), the most prevalent sustained cardiac arrhythmia, imposes a substantial global health burden through its associations with stroke, heart failure, and all-cause mortality. Although hypertension remains a well-documented modifiable risk factor for AF,^1^, ^2^, ^3^ growing evidence challenges the sufficiency of conventional seated blood pressure (BP) measurements for comprehensive risk assessment.^4^^,^^5^ This limitation is particularly evident in individuals with masked hypertension or positional BP variability, where supine measurements may uncover critical hemodynamic profiles.

Recent advances in BP phenotyping revealed that longitudinal trajectories and temporal variability in systolic BP (SBP) independently predict AF risk.^4^ Mendelian randomization studies further demonstrated a causal relationship between genetically elevated BP and AF susceptibility, with each 10 mmHg increase in SBP conferring 19% higher AF risk.^3^^,^^6^ However, current risk stratification paradigms predominantly rely on seated BP measurements, potentially overlooking the prognostic significance of supine hypertension, a phenomenon associated with autonomic dysfunction and altered nocturnal hemodynamics. Notably, a recent Atherosclerosis Risk in Communities study showed that supine hypertension was independently associated with adverse cardiovascular outcomes, including coronary heart disease, stroke, and mortality, even in the absence of seated hypertension.^7^ However, its potential relationship with AF was not assessed in that study.^7^

To address this gap, we investigated both seated and supine BP measurements in a large, well-characterized cohort from the UK Biobank. By examining interaction effects across age groups and cardiovascular risk profiles, our study aims to determine whether supine BP provides incremental prognostic value beyond seated measurements, thereby informing more precise and individualized hypertension management strategies for AF prevention.

## Methods

### Study population

**Figure 1:**
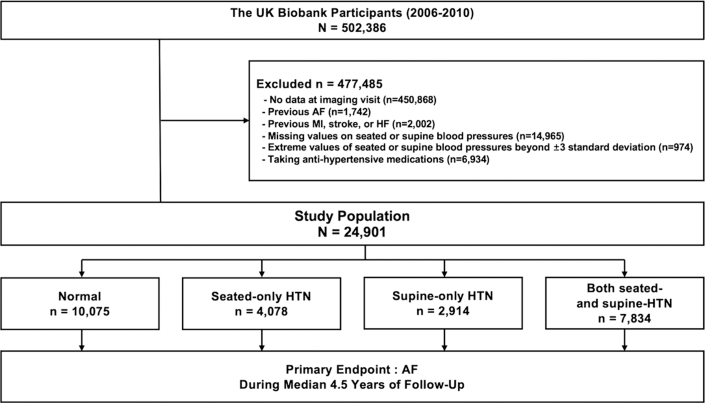
Study Flow The flow diagram of the present study is shown. AF = atrial fibrillation; HF = heart failure; HTN = hypertension; MI = myocardial infarction.

This study was conducted using data from the UK Biobank, a large-scale, prospective longitudinal cohort of individuals registered with the UK National Health Service and followed since 2006. Detailed information regarding the study protocol of the UK Biobank has been described in a previous publication.^8^ From the initial cohort of 502,386 participants, a total of 477,485 were excluded based on predefined criteria, resulting in a final study population of 24,901 participants (Figure 1). The exclusion criteria were as follows: 1) lack of data from the imaging visit; 2) history of AF; 3) history of myocardial infarction, stroke, or heart failure; 4) missing values for seated or supine BP measurements; 5) extreme seated or supine BP values beyond ±3 SDs; and 6) use of antihypertensive medications. Participants were categorized into 4 groups based on the presence of seated and/or supine hypertension. The normal group included individuals without seated or supine hypertension (n = 10,075); the seated-only hypertension group included those with seated hypertension but not supine hypertension (n = 4,078); the supine-only hypertension group included those with supine hypertension but not seated hypertension (n = 2,914); and the rest were the both seated- and supine-hypertension group (n = 7,834). Ethical approval for this research was obtained from the Northwest Multi-Center Research Ethics Committee and sanctioned by the UK Biobank’s review committee (application number 76593). The study was also approved by the National Health Service National Research Ethics Service on June 17, 2011 (Ref 11/NW/0382), with an extension granted on May 10, 2016 (Ref 16/NW/0274). All participants provided informed consent.

### Definition of hypertension, covariates, and outcomes

Detailed information on covariates and outcomes is provided in Supplemental Table 1. Hypertension was operationally defined as an SBP ≥140 mm Hg or a diastolic BP (DBP) ≥90 mm Hg based on measurements obtained at the imaging visit and does not represent a clinical diagnosis of hypertension. Seated BP was measured using the Omron 705 IT electronic sphygmomanometer according to the standardized UK Biobank protocol. Participants were seated with their back supported and feet flat on the floor; an appropriately sized cuff was selected based on mid-upper arm circumference. Two measurements were taken from the left arm, with at least 1 minute of rest between readings, and the arm was supported at heart level. If the electronic device was unavailable, a manual sphygmomanometer and stethoscope were used. The average of the 2 readings was used for analysis.

Supine BP was measured during the imaging visit using the Vicorder device. Participants were positioned in a supine posture, and a brachial cuff was applied to the left arm with proper sizing and placement verified by trained staff. The Vicorder device measured brachial BP and pulse wave velocity to evaluate central hemodynamics, in conjunction with MRI imaging for assessing aortic distensibility and vascular stiffness. Following the completion of the standard physical examination and seated measurements, participants moved to the imaging workstation where, after an appropriate period of postural transition and rest, supine BP was measured during the imaging visit using the Vicorder device.

**Figure 2:**
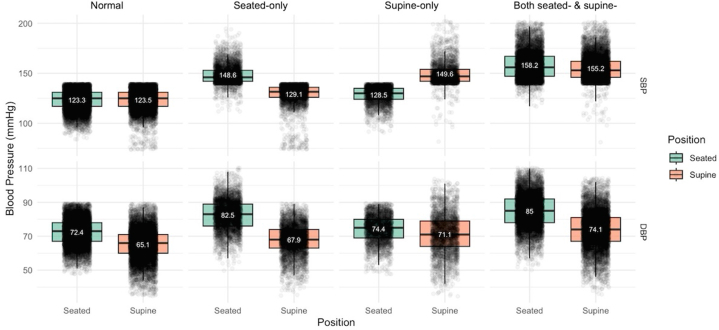
Blood Pressure Distribution Across Hypertension Categories and Measurement Positions Boxplots with overlaid dot plots show the distribution of seated and supine systolic blood pressure (SBP) and diastolic blood pressure (DBP) across 4 hypertension groups: normal, seated-only hypertension, supine-only hypertension, and both seated and supine hypertension. The mean values are annotated within each box.

The standardized protocols for both seated and supine BP measurements are available on the UK Biobank website. The distributions of SBP and DBP for each group were presented in Figure 2.

The primary outcome was the incidence of AF, identified using the International Classification of Diseases, Tenth Revision code I48 registered in the inpatient, outpatient, or death registry incorporated in the UK Biobank outcome database (field ID 41270, 41280, and 131350). The median follow-up duration was 4.5 years (IQR: 3.6-6.4 years). The overall study design, cohort grouping, and principal findings are summarized in the Central Illustration.

**Central Illustration:**
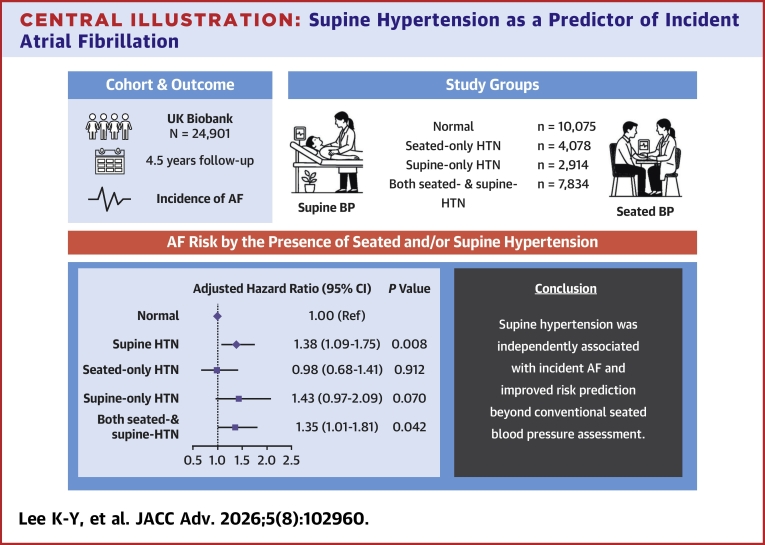
Supine Hypertension as a Predictor of Incident Atrial Fibrillation This central illustration summarizes the association between seated and supine hypertension phenotypes and incident atrial fibrillation (AF) in 24,901 UK Biobank participants without prior cardiovascular disease or antihypertensive treatment. Supine hypertension was independently associated with incident AF and improved AF risk prediction beyond conventional seated blood pressure assessment. AF = atrial fibrillation; BP = blood pressure; HTN = hypertension.

### Statistical analyses

Continuous variables were expressed as means with SDs or medians with IQRs, whereas categorical variables were summarized using frequencies and percentages. Group comparisons for categorical data were conducted using the chi-square test, and continuous data were compared across the 4 groups using one-way analysis of variance. The Kaplan-Meier method was employed to estimate the cumulative incidence of AF, and differences between groups were evaluated using the log-rank test. Multivariable Cox proportional hazards regression models were used to explore the adjusted associations between hypertension status and incident AF, yielding HRs and 95% CIs. In the Cox proportional hazards regression analysis, an unadjusted model and 2 multivariable-adjusted models were constructed. Model 1 was adjusted for age and sex. Model 2 was further adjusted for ethnicity, body mass index, smoking status, alcohol consumption, diabetes mellitus, dyslipidemia, and resting heart rate, in addition to the covariates in model 1. Participants with missing data in variables included in each model were excluded from the corresponding analysis. The proportional hazards assumption was assessed using Schoenfeld residuals and was not violated (global *P* = 0.87). To evaluate the incremental predictive value of seated, supine, and combined hypertension for AF occurrence, we calculated Harrell C-index and the category-free net reclassification improvement (NRI). Statistical significance was defined as a 2-sided *P* value <0.05. All analyses were conducted using R software (version 4.4.3, R Foundation for Statistical Computing).

## Results

### Baseline characteristics of the study population

The baseline characteristics of the study population are shown in Table 1. Among 24,901 participants, the mean age was 63.1 ± 7.7 years, 44.0% were males, 16.4% had seated-only hypertension, 11.7% had supine-only hypertension, and 31.5% had both seated and supine hypertension. Participants with both seated and supine hypertension were significantly older (mean age 66.0 ± 7.2 years), had a higher body mass index (26.7 ± 4.3 kg/m^2^), and showed a greater prevalence of diabetes mellitus (3.5%) and dyslipidemia (19.8%) compared to the other groups. The seated-only group had a higher proportion of males (60.1%), whereas the supine-only group appeared to be more female predominant (29.2% male). Resting heart rate was higher in both the supine-only (70.7 ± 11.2 bpm) and the both seated and supine hypertension group (69.5 ± 12.0 bpm) compared to the normal group (66.8 ± 10.9 bpm). The distribution of SBP and DBP measurements across different hypertension phenotypes and measurement positions (seated vs supine) is illustrated in Figure 2 and Supplemental Figure 1, providing visual confirmation of the overlap and discrepancies among the groups.

**Table 1:** Baseline Characteristics

	Total (N = 24,901)	Normal (n = 10,075)	Seated-Only HTN (n = 4,078)	Supine-Only HTN (n = 2,914)	Both Seated and Supine-HTN (n = 7,834)	*P* Value
Age, years	63.1 ± 7.7	60.6 ± 7.4	63.7 ± 7.7	63.3 ± 7.3	66.0 ± 7.2	<0.001
Male, n (%)	10,950 (44.0)	4,117 (40.9)	2,449 (60.1)	851 (29.2)	3,533 (45.1)	<0.001
Ethnicity, n (%)						0.005
White	22,836 (91.7)	9,171 (91.0)	3,757 (92.1)	2,666 (91.5)	7,242 (92.4)	
Asian	884 (3.6)	394 (3.9)	142 (3.5)	122 (4.2)	226 (2.9)	
Black	695 (2.8)	292 (2.9)	113 (2.8)	69 (2.4)	221 (2.8)	
Mixed	295 (1.2)	124 (1.2)	44 (1.1)	39 (1.3)	88 (1.1)	
Others	182 (0.7)	88 (0.9)	21 (0.5)	16 (0.5)	57 (0.7)	
Body mass index, kg/m^2^	25.9 ± 4.1	25.1 ± 3.7	26.3 ± 4.0	25.9 ± 4.2	26.7 ± 4.3	<0.001
Smoking status, n (%)						<0.001
Nonsmoker	15,932 (64.0)	6,559 (65.1)	2,609 (64.0)	1,872 (64.2)	4,892 (62.4)	
Ex-smoker	7,879 (31.6)	3,068 (30.5)	1,301 (31.9)	899 (30.9)	2,611 (33.3)	
Current smoker	865 (3.5)	386 (3.8)	133 (3.3)	112 (3.8)	234 (3.0)	
Alcohol consumption, n (%)						<0.001
Never	773 (3.1)	297 (2.9)	102 (2.5)	115 (3.9)	259 (3.3)	
Previous	769 (3.1)	340 (3.4)	112 (2.7)	104 (3.6)	213 (2.7)	
Current	23,190 (93.1)	9,390 (93.2)	3,837 (94.1)	2,672 (91.7)	7,291 (93.1)	
Diabetes mellitus, n (%)	737 (3.0)	254 (2.5)	131 (3.2)	78 (2.7)	274 (3.5)	<0.001
Dyslipidemia, n (%)	3,924 (15.8)	1,229 (12.2)	699 (17.1)	446 (15.3)	1,550 (19.8)	<0.001
Seated SBP, mm Hg	139.0 ± 19.1	123.3 ± 10.1	148.6 ± 9.3	128.5 ± 8.5	158.2 ± 13.7	<0.001
Seated DBP, mm Hg	78.2 ± 10.4	72.4 ± 7.7	82.5 ± 8.9	74.4 ± 7.9	85.0 ± 9.9	<0.001
Supine SBP, mm Hg	137.5 ± 18.1	123.5 ± 10.0	129.1 ± 10.4	149.6 ± 11.2	155.2 ± 12.4	<0.001
Supine DBP, mm Hg	69.1 ± 10.7	65.1 ± 9.1	67.9 ± 9.0	71.1 ± 11.6	74.1 ± 11.0	<0.001
Resting heart rate, bpm	68.2 ± 11.6	66.8 ± 10.9	67.0 ± 11.9	70.7 ± 11.2	69.5 ± 12.0	<0.001

Values are mean ± SD or n (%).

DBP = diastolic blood pressure; HTN = hypertension; SBP = systolic blood pressure.

### Association of blood pressure and incident atrial fibrillation

The risk of incident AF according to seated and supine BP measurements is presented in Table 2. A 10-mm Hg increase in seated SBP was significantly associated with an 8% higher risk of incident AF (adjusted HR [aHR]: 1.08; 95% CI: 1.01-1.15; *P* = 0.024), whereas seated DBP was not significantly associated with AF risk. Similarly, a 10-mm Hg increase in supine SBP was associated with a 12% higher risk of incident AF (aHR: 1.12; 95% CI: 1.05-1.20; *P* = 0.001), whereas supine DBP showed no significant association with AF risk.

**Table 2:** Incidence and Risk of Atrial Fibrillation According to Seated and Supine Blood Pressure Measurements

	Unadjusted HR (95% CI)	*P* Value	Adjusted HR (M1) (95% CI)	*P* Value	Adjusted HR (M2) (95% CI)	*P* Value
Seated SBP, per 10-mm Hg increase	1.19 (1.13-1.26)	<0.001	1.08 (1.02-1.15)	0.016	1.08 (1.01-1.15)	0.024
Seated DBP, per 10-mm Hg increase	1.05 (0.94-1.17)	0.373	1.01 (0.91-1.13)	0.839	0.99 (0.88-1.12)	0.876
Supine SBP, per 10-mm Hg increase	1.17 (1.11-1.25)	<0.001	1.12 (1.05-1.20)	0.001	1.12 (1.05-1.20)	0.001
Supine DBP, per 10-mm Hg increase	1.13 (1.02-1.26)	0.019	1.06 (0.95-1.18)	0.324	1.06 (0.95-1.19)	0.299

Adjusted HR was calculated in the multivariable Cox regression model.

Model 1: Adjusted for age and sex.

Model 2: Adjusted for age, sex, BMI, smoking status, alcohol consumption, ethnicity, history of diabetes mellitus, dyslipidemia, and resting heart rate.

Abbreviations as in Table 1.

### The risk of incident atrial fibrillation according to seated and supine hypertension

**Figure 3:**
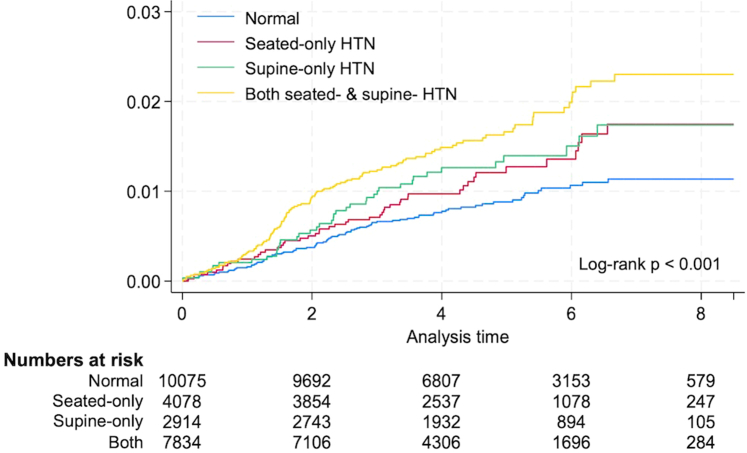
Kaplan-Meier Curve for Incidence Atrial Fibrillation by Hypertension Status Abbreviation as in Figure 1.

The risk of incident AF according to seated and supine hypertension groups is presented in Table 3. When participants were categorized based on the presence of seated or supine hypertension, supine hypertension was significantly associated with an increased risk of AF (aHR: 1.38; 95% CI: 1.09-1.75; *P* = 0.008). When participants were categorized into 4 groups, those with both seated and supine hypertension had a significantly increased risk of incident AF (aHR: 1.35; 95% CI: 1.01-1.81; *P* = 0.042), whereas the supine-only hypertension group showed a similar trend with marginal significance (aHR: 1.43; 95% CI: 0.97-2.09; *P* = 0.070). The Kaplan-Meier curve showed that participants with either seated-only hypertension or supine-only hypertension had a higher risk of incident AF compared to the normal group, with the highest risk observed in those with both seated and supine hypertension (log-rank *P* < 0.001) (Figure 3).

**Table 3:** Incidence and Risk of Atrial Fibrillation According to Seated and Supine Hypertension

	N	Event	IR, per 1000 PY	Unadjusted HR		Adjusted HR (M1)		Adjusted HR (M2)	
				HR (95% CI)	*P* Value	HR (95% CI)	*P* Value	HR (95% CI)	*P* Value
Normal	12,989	128	1.95	1 (Ref)	-	1 (Ref)	-	1 (Ref)	-
Seated HTN	11,912	171	3.11	1.57 (1.25-1.98)	<0.001	1.12 (0.89-1.42)	0.332	1.10 (0.86-1.40)	0.439
Normal	14,153	137	1.94	1 (Ref)	-	1 (Ref)	-	1 (Ref)	-
Supine HTN	10,748	162	3.24	1.65 (1.31-2.07)	<0.001	1.38 (1.09-1.74)	0.007	1.38 (1.09-1.75)	0.008
Normal	10,075	89	1.74	1 (Ref)	-	1 (Ref)	-	1 (Ref)	-
Seated-only HTN	4,078	48	2.45	1.39 (0.98-1.98)	0.065	1.00 (0.70-1.43)	0.999	0.98 (0.68-1.41)	0.912
Supine-only HTN	2,914	39	2.67	1.53 (1.05-2.22)	0.028	1.41 (0.96-2.06)	0.078	1.43 (0.97-2.09)	0.070
Both seated and supine HTN	7,834	123	3.47	1.95 (1.49-2.57)	<0.001	1.36 (1.03-1.81)	0.031	1.35 (1.01-1.81)	0.042

Adjusted HR was calculated in the multivariable Cox regression model.

Model 1: Adjusted for age and sex.

Model 2: Adjusted for age, sex, BMI, smoking status, alcohol consumption, ethnicity, history of diabetes mellitus, dyslipidemia, and resting heart rate.

IR = incidence rate; PY = person-year; other abbreviation as in Table 1.

The incidence and risk of AF according to isolated systolic and diastolic hypertension, stratified by measurement position, are presented in Table 4. When BP was measured in the seated position, neither isolated systolic nor diastolic hypertension, nor combined hypertension, was significantly associated with incident AF. However, when measured in the supine position, isolated systolic hypertension was significantly associated with incident AF (aHR: 1.38; 95% CI: 1.08-1.75; *P* = 0.010). Although the incidence rate of AF was comparable between those with isolated systolic hypertension and those with both systolic and diastolic hypertension (3.22 vs 3.96 per 1,000 person-years, respectively), only isolated systolic hypertension demonstrated a statistically significant association, whereas the association for combined hypertension did not reach statistical significance (aHR: 1.71; 95% CI: 0.94-3.11; *P* = 0.078).

**Table 4:** Incidence and Risk of Atrial Fibrillation According to Isolated Systolic and Diastolic Hypertension, Stratified by Measurement Position

	N	Event	IR, per 1000 PY	Unadjusted HR		Adjusted^a^ HR (M1)		Adjusted HR (M2)	
				HR (95% CI)	*P* Value	HR (95% CI)	*P* Value	HR (95% CI)	*P* Value
Seated Position									
Normal	12,989	128	1.95	1 (Ref)		1 (Ref)		1 (Ref)	
Isolated systolic HTN	8,368	120	3.13	1.58 (1.23-2.02)	<0.001	1.08 (0.83-1.39)	0.573	1.08 (0.83-1.40)	0.573
Isolated diastolic HTN	372	5	2.61	1.35 (0.55-3.29)	0.513	1.50 (0.61-3.67)	0.376	1.46 (0.59-3.59)	0.408
Systolic and diastolic HTN	3,172	46	3.12	1.58 (1.13-2.22)	0.007	1.22 (0.87-1.71)	0.26	1.21 (0.85-1.70)	0.288
Supine position									
Normal	14,153	137	1.94	1 (Ref)		1 (Ref)		1 (Ref)	
Isolated systolic HTN	9,970	149	3.22	1.64 (1.30-2.07)	<0.001	1.36 (1.08-1.73)	0.01	1.38 (1.08-1.75)	0.010
Isolated diastolic HTN	147	1	1.29	0.67 (0.09-4.77)	0.687	0.62 (0.09-4.41)	0.63	0.63 (0.09-4.51)	0.645
Systolic and diastolic HTN	631	12	3.96	2.03 (1.13-3.66)	0.019	1.70 (0.94-3.07)	0.077	1.71 (0.94-3.11)	0.078

Model 1: Adjusted for age and sex.

Model 2: Adjusted for age, sex, BMI, smoking status, alcohol consumption, ethnicity, history of diabetes mellitus, dyslipidemia, and resting heart rate.

Abbreviations as in Tables 1 and 3.

a Adjusted HR was calculated in the multivariable Cox regression model.

### Additive predictive value of seated and supine hypertension on AF prediction

The additive predictive value of seated and supine hypertension for the risk of AF is presented in Table 5. Model 1 included seated hypertension; model 2 included supine hypertension; and model 3 combined both seated and supine hypertension. Using model 1 as the reference, Harrell C-index significantly improved with the addition of supine hypertension to seated hypertension (0.553 vs 0.576; *P* = 0.033). Furthermore, the category-free NRI showed a significant improvement of reclassification effect with the addition of supine hypertension (22.3% [10.9-33.7]; *P* < 0.001).

**Table 5:** Additive Value of Supine Hypertension on Predicting Atrial Fibrillation

	Harrell's C-index (95% CI)	*P* Value	Category-free NRI (95% CI)	*P* Value
M1: seated HTN	0.553 (0.524-0.582)	(Ref)	(Ref)	-
M2: supine HTN	0.563 (0.534-0.592)	0.528	-	-
M3: M1 + M2	0.576 (0.545-0.608)	0.033	22.3% (10.9%-33.7%)	<0.001

Model 1: seated hypertension.

Model 2: supine hypertension.

Model 3: combined seated and supine hypertension.

NRI = net reclassification improvement; other abbreviation as in Table 1.

### Sensitivity analysis

In the subgroup analysis stratified by age, participants younger than 65 years with supine-only hypertension or both seated and supine hypertension had a significantly higher risk of incident AF compared to the normotensive group (Table 6). Among participants aged <65 years, supine-only hypertension was associated with an aHR of 2.66 (95% CI: 1.53-4.64; *P* = 0.001), and both seated and supine hypertension with an aHR of 2.08 (95% CI: 1.29-3.35; *P* = 0.003). In contrast, among participants aged ≥65 years, none of the hypertension patterns, including seated-only, supine-only, or both seated and supine hypertension, showed a statistically significant association with AF risk. Subgroup analyses according to sex, obesity, diabetes mellitus, and dyslipidemia revealed no statistically significant interactions.

**Table 6:** Subgroup Analysis for Incidence and Risk of Atrial Fibrillation According to Seated and Supine Hypertension

Subgroup	Groups	Case	Event	IR, per 1000 PY	HR (95% CI)	*P* Value	*P* Value for interaction
Age							
<65 years	Normal	7,081	35	0.947	1.00 (Ref)		<0.001
	Seated-only HTN	2,217	10	0.885	0.74 (0.36-1.50)	0.401	
	Supine-only HTN	1,654	21	2.44	2.66 (1.53-4.64)	0.001	
	Both seated-and supine-HTN	3,344	42	2.57	2.08 (1.29-3.35)	0.003	
≥65 years	Normal	2,994	54	3.82	1.00 (Ref)		
	Seated-only HTN	1,861	38	4.56	1.04 (0.68-1.59)	0.852	
	Supine-only HTN	1,260	18	2.99	0.84 (0.49-1.45)	0.539	
	Both seated-and supine-HTN	4,490	81	4.24	1.06 (0.74-1.50)	0.764	
Sex							
Male	Normal	4,117	58	2.82	1.00 (Ref)		0.6246
	Seated-only HTN	2,449	35	2.96	0.92 (0.60-1.41)	0.692	
	Supine-only HTN	851	17	4.03	1.24 (0.72-2.14)	0.444	
	Both seated and supine HTN	3,533	78	4.85	1.33 (0.93-1.90)	0.120	
Female	Normal	5,958	31	1.02	1.00 (Ref)		
	Seated-only HTN	1,629	13	1.67	1.20 (0.62-2.31)	0.588	
	Supine-only HTN	2,063	22	2.12	1.67 (0.96-2.91)	0.071	
	Both seated and supine HTN	4,301	45	2.33	1.42 (0.87-2.30)	0.161	
Obesity							
BMI <30	Normal	9,103	73	1.59	1.00 (Ref)		0.5208
	Seated-only HTN	3,490	41	2.46	1.10 (0.74-1.63)	0.644	
	Supine-only HTN	2,495	31	2.48	1.43 (0.93-2.19)	0.101	
	Both seated and supine HTN	6,472	93	3.19	1.38 (1.00-1.91)	0.049	
BMI ≥30	Normal	962	16	3.17	1.00 (Ref)		
	Seated-only HTN	584	7	2.39	0.65 (0.26-1.61)	0.347	
	Supine-only HTN	413	8	3.86	1.37 (0.57-3.32)	0.481	
	Both seated and supine HTN	1,355	30	4.82	1.26 (0.66-2.41)	0.491	
Diabetes mellitus							
No	Normal	9,821	84	1.69	1.00 (Ref)		0.255
	Seated-only HTN	3,947	46	2.42	1.01 (0.70-1.46)	0.974	
	Supine-only HTN	2,836	39	2.74	1.48 (1.00-2.18)	0.047	
	Both seated and supine HTN	7,560	116	3.39	1.36 (1.01-1.83)	0.041	
Yes	Normal	254	5	4.03	1.00 (Ref)		
	Seated-only HTN	131	2	3.23	0.86 (0.16-4.81)	0.868	
	Supine-only HTN	78	0	0	0.00 (0.00-Inf)	0.998	
	Both seated and supine HTN	274	7	5.72	1.55 (0.44-5.49)	0.494	
Dyslipidemia							
No	Normal	8,846	70	1.56	1.00 (Ref)		0.1923
	Seated-only HTN	3,379	37	2.26	1.02 (0.68-1.53)	0.929	
	Supine-only HTN	2,468	32	2.6	1.55 (1.01-2.37)	0.045	
	Both seated and supine HTN	6,284	100	3.5	1.53 (1.11-2.11)	0.010	
Yes	Normal	1,229	19	3.14	1.00 (Ref)		
	Seated-only HTN	699	11	3.37	0.88 (0.41-1.88)	0.736	
	Supine-only HTN	446	7	3.05	0.96 (0.40-2.33)	0.933	
	Both seated and supine HTN	1,550	23	3.35	0.88 (0.47-1.67)	0.703	

BMI = body mass index; other abbreviations as in Tables 1 and 3.

In sensitivity analyses using a hypertension threshold of ≥130/80 mm Hg (Supplemental Table 2), the overall pattern of associations was broadly consistent with the primary analysis. When categorized into seated and supine hypertension groups, supine hypertension remained significantly associated with incident AF after multivariable adjustment (aHR: 1.35; 95% CI: 1.06-1.71; *P* = 0.014), whereas seated hypertension was not (aHR: 0.99; 95% CI: 0.77-1.26; *P* = 0.905). However, when participants were further classified into 4 groups (normal, seated-only hypertension, supine-only hypertension, and both), none of the categories showed a statistically significant association with incident AF.

## Discussions

In this large cohort study utilizing data from the UK Biobank, we demonstrated the main findings as follows: 1) seated and supine SBP, but not DBP, were significantly associated with an increased risk of AF; 2) the association between supine hypertension and incident AF was stronger than that observed in seated hypertension; 3) the combination of both measurements improved predictive performance for AF; and 4) in participants aged <65 years, supine-only or combined seated- and supine-hypertension were significantly associated with increased AF risk, whereas no such associations were observed in those aged ≥65 years. These findings suggest that supine BP assessment may reveal a subset of individuals at elevated risk for AF who may be missed by conventional seated BP measurements alone.

Our results are consistent with prior evidence indicating that nontraditional BP phenotypes, such as masked or nocturnal hypertension, are important predictors of AF.^9^^,^^10^ The present study extends the knowledge by highlighting the incremental prognostic value of supine BP, particularly in identifying individuals with supine hypertension or autonomic dysfunction. Importantly, although supine hypertension alone is associated with increased AF risk,^11^ its prognostic value may reflect underlying autonomic dysfunction or masked hypertension not captured by seated BP, although these mechanisms were not directly assessed and remain speculative.^12^ We additionally note that the study population in UK Biobank is unlikely to represent individuals with overt autonomic failure syndromes, and the mechanistic interpretation linking supine BP to AF via autonomic pathways should be regarded as speculative given that autonomic function was not directly measured in this study. These mechanisms may be associated with atrial remodeling and increased arrhythmia susceptibility, although this remains speculative.^13^^,^^14^ Recognizing these nontraditional BP phenotypes through supine measurements may help identify at-risk individuals who might otherwise be misclassified as normotensive. In our results, the value of supine hypertension was further supported by improvements in Harrell C-index and the category-free NRI when supine BP was added to models based solely on seated BP. These results indicate that supine BP may serve as an adjunctive physiological marker that refines AF risk recognition beyond conventional seated measurements, rather than as a stand-alone calibrated prognostic tool.

The discordance between supine and seated hypertension observed in our study may be explained by differences in autonomic regulation between supine and seated positions. Seated measurements are more influenced by gravitational pooling of blood in the lower extremities, which activates sympathetic tone to maintain perfusion. In contrast, supine measurements are less affected by gravitational shifts and may more directly reflect baseline sympathetic overactivity, especially in individuals with preserved autonomic function. Regarding the inclusion of resting heart rate in our multivariable models, its adjustment may have partially attenuated the estimated autonomic pathway linking supine BP to AF. Nevertheless, the persistent significance of supine BP parameters in the fully adjusted model indicates that supine BP captures hemodynamic information beyond autonomic fluctuations alone, potentially reflecting increased central venous return, atrial stretch, and structural remodeling that independently contribute to arrhythmogenesis. With respect to potential collinearity, in an additional analysis simultaneously including both seated and supine hypertension in the same multivariable model, supine hypertension remained independently associated with incident AF (HR: 1.25; 95% CI: 1.02-1.54; *P* = 0.036), whereas seated hypertension was not (HR: 0.95; 95% CI: 0.77-1.18; *P* = 0.654); variance inflation factors were well within acceptable thresholds (variance inflation factor range 1.30-1.33), confirming that multicollinearity did not meaningfully distort our HR estimates. This phenomenon has been described in patients with autonomic dysfunction, in whom supine hypertension occurs despite normotension or even hypotension in upright positions.^15^^,^^16^ These physiologic differences underscore the importance of considering supine BP measurements when assessing cardiovascular risk, particularly in younger individuals who may exhibit stronger sympathetic responses in the supine position.

Our subgroup analyses revealed that the association between supine hypertension, either isolated or combined with seated hypertension, and AF was particularly pronounced among individuals younger than 65 years. This observation may reflect increased susceptibility to hemodynamic changes driven by autonomic dysregulation, which tends to be more pronounced in younger individuals with preserved autonomic responsiveness.^17^^,^^18^ Supine hypertension has been linked to increased sympathetic drive, particularly in populations without marked autonomic failure, and may indicate impaired baroreflex buffering or neurohumoral imbalance.^19^ In younger individuals, heightened autonomic nervous system activity can predispose to arrhythmias such as AF.^20^ In addition, the higher prevalence of vagally mediated AF in younger patients supports a distinct pathophysiological mechanism rooted in autonomic imbalance.^17^ Collectively, these factors underscore the importance of accounting for age-related differences in autonomic function when evaluating the AF risk associated with supine hypertension. In contrast, no significant associations were observed among older individuals or in subgroups stratified by sex, obesity, diabetes, or dyslipidemia, suggesting that age may modify the relationship between supine hypertension and AF risk.

In sensitivity analyses using a lower hypertension threshold (≥130/80 mm Hg), the overall pattern of associations was broadly consistent with the primary findings. Notably, supine hypertension remained significantly associated with incident AF after multivariable adjustment, whereas seated hypertension did not. Although analyses using 4 hypertension categories did not reach statistical significance, the direction of the associations was generally preserved. These findings suggest that the association between supine BP and AF risk may be relatively consistent across different hypertension definitions, whereas the association with seated BP appears more sensitive to threshold selection. The attenuation of statistical significance in the four-category analysis may be partly explained by reduced statistical power and greater overlap between groups under the lower threshold.

### Study limitations

Several limitations merit consideration. First, BP was measured at a single time point, which may not capture long-term variability or account for potential white-coat effects. Second, out-of-office BP measurements (eg, ambulatory or home BP) were not available in the UK Biobank imaging data set, which may limit a more comprehensive assessment of BP patterns. In addition, we could not directly compare or integrate supine BP with established clinical AF risk scores, such as CHARGE-AF, due to a lack of concurrent baseline data for all score components during the imaging visit. Future studies should evaluate whether incorporating supine BP into these validated prediction models improves their predictive performance. Third, AF ascertainment was based on International Classification of Diseases codes, which may not fully capture paroxysmal or subclinical AF. Fourth, the relatively short follow-up duration may limit the detection of long-term associations. Fifth, the study population consisted of relatively healthy volunteers from the UK Biobank, which may limit the generalizability of our findings to higher-risk or more ethnically diverse populations. Finally, despite comprehensive multivariable adjustment, residual confounding by unmeasured factors, including sleep apnea, autonomic dysfunction, and physical activity, cannot be fully excluded.

## Conclusions

Supine hypertension was significantly associated with incident AF, particularly among younger individuals. Supine BP may capture prognostic information not fully reflected by conventional seated BP measurements.

Perspectives **COMPETENCY IN MEDICAL KNOWLEDGE:** Hypertension is a major modifiable risk factor for AF, yet its assessment is largely based on seated BP measurements. This study demonstrates that supine BP provides incremental prognostic information for incident AF beyond conventional seated measurements. Incorporating supine BP assessment may improve identification of patients at increased arrhythmic risk who could benefit from earlier preventive strategies.

**TRANSLATIONAL OUTLOOK:** Routine assessment of supine BP is not currently standardized in clinical practice, which may limit immediate implementation. Future studies are needed to determine optimal measurement protocols, clarify underlying autonomic or hemodynamic mechanisms, and evaluate whether targeting supine hypertension can reduce AF incidence.

## Funding support and author disclosures

This research was supported by a grant from the Patient-Centered Clinical Research Coordinating Center (PACEN) funded by the Ministry of Health & Welfare, Republic of Korea (grant number: RS-2021-KH119931), by the 10.13039/501100003725National Research Foundation of Korea (NRF) grant funded by the Korea government (MSIT) (IRIS RS-2024-00340590), and by Yuhan Corporation (IIT 076). Dr Eue-Keun Choi reports research grants or speaking fees from Bayer, BMS/Pfizer, Biosense Webster, Chong Kun Dang, Daiichi-Sankyo, Dreamtech Co., Ltd., Medtronic, Samjinpharm, Sanofi-Aventis, Seers Technology, Skylabs, and Yuhan. Dr Lip served as consultant and speaker for BMS/Pfizer, Boehringer Ingelheim, Anthos and Daiichi-Sankyo, but no fees are received personally; no other relationships or activities that could appear to have influenced the submitted work. All other authors have reported that they have no relationships relevant to the contents of this paper to disclose.
