# Supplementary material for: Supine Hypertension as a Predictor of Atrial Fibrillation: A UK Biobank Cohort Study
Source: JACC Adv. 2026 Jul 9;5(8):102960. doi: 10.1016/j.jacadv.2026.102960 (PMC13377151; doi:10.1016/j.jacadv.2026.102960)
Supplement: Supplemental Material [file mmc1.pdf]

Supplemental material online

Supine hypertension as a Predictor of Atrial Fibrillation: A UK Biobank Cohort Study

Table of Contents

1. Supplemental Tables

Supplemental Table 1. Definitions of covariates and outcomes

Supplemental Table 2. Incidence of atrial fibrillation by seated and supine hypertension defined as ≥130/80 mmHg

2. Supplemental Figure

Supplemental Figure 1. Scatter plot of Systolic and Diastolic Blood Pressure Across Measurement Positions

Supplemental Table 1. Definitions of covariates and outcomes

Covariates	UK Biobank Field ID	Code
Age	21022	
Sex	31	
Ethnicity	21000	
Body mass index	21001	
Current smoking	20116	
Alcohol consumption	1558, 20117	
Seated SBP	4080, 93	
Seated DBP	4079, 94	
Supine SBP	12697	
Supine DBP	12698	
Resting heart rate	102	
Diabetes mellitus	2443, 6153, 6177, 20002, 130708, 130710, 130712, 130714, 41270, 41280	E11-E14
Hypertension on medication	6153, 6177, 20002, 2966, 131286, 131288, 131290, 131292, 131294, 41270, 41280	I10-I13, I15
Dyslipidemia	6153, 6177, 20002, 130814, 41270, 41280	E78
Previous MI	20002, 41270, 41280, 131298, 131300, 131302, 42000	1075, I21-I23
Previous HF	20002, 41270, 41280, 131354	1076, I50, I110, I130, I132
Previous stroke	20002, 6150, 41270, 41280, 131360, 131362, 131364, 131366, 131368, 42006	1081, I60-I64
Previous AF	20002, 41270, 41280, 131350	1471, I48
**Outcomes**	**UK Biobank Field ID**	**Code**
Atrial fibrillation	41270, 41280, 131350	I48

Supplemental Table 2. Incidence of atrial fibrillation by seated and supine hypertension defined as ≥130/80 mmHg

	N	Event	IR, per 1000 PY	Unadjusted HR		Adjusted HR (M1)		Adjusted HR (M2)	
				HR (95% CI)	*P*	HR (95% CI)	*P*	HR (95% CI)	*P*
**Normal**	7272	69	1.85	1 (Ref)		1 (Ref)		1 (Ref)	
**Seated HTN**	17629	230	2.75	1.47(1.12 - 1.92)	0.005	1.06 (0.83 - 1.34)	0.649	0.99 (0.77 - 1.26)	0.905
**Normal**	8315	66	1.56	1 (Ref)		1 (Ref)		1 (Ref)	
**Supine HTN**	16586	233	2.97	1.88 (1.43 - 2.47)	<0.001	1.38 (1.09 - 1.74)	0.007	1.35 (1.06 - 1.71)	0.014
**Normal**	4896	44	1.76	1 (Ref)		1 (Ref)		1 (Ref)	
**Seated-only HTN**	4476	48	2.15	0.98 (0.60 - 1.59)	0.922	0.73 (0.45 - 1.19)	0.207	0.82 (0.54 - 1.25)	0.361
**Supine-only HTN**	3098	42	2.66	1.47 (0.91 - 2.36)	0.112	1.35 (0.84 - 2.16)	0.22	1.32 (0.86 - 2.02)	0.205
**Both seated-and supine-HTN**	19365	318	3.55	1.94 (1.36 - 2.75)	<0.001	1.30 (0.91 - 1.86)	0.152	1.19 (0.86 - 1.66)	0.291

Supplemental Figure 1. Scatter plot of Systolic and Diastolic Blood Pressure Across Measurement Positions.

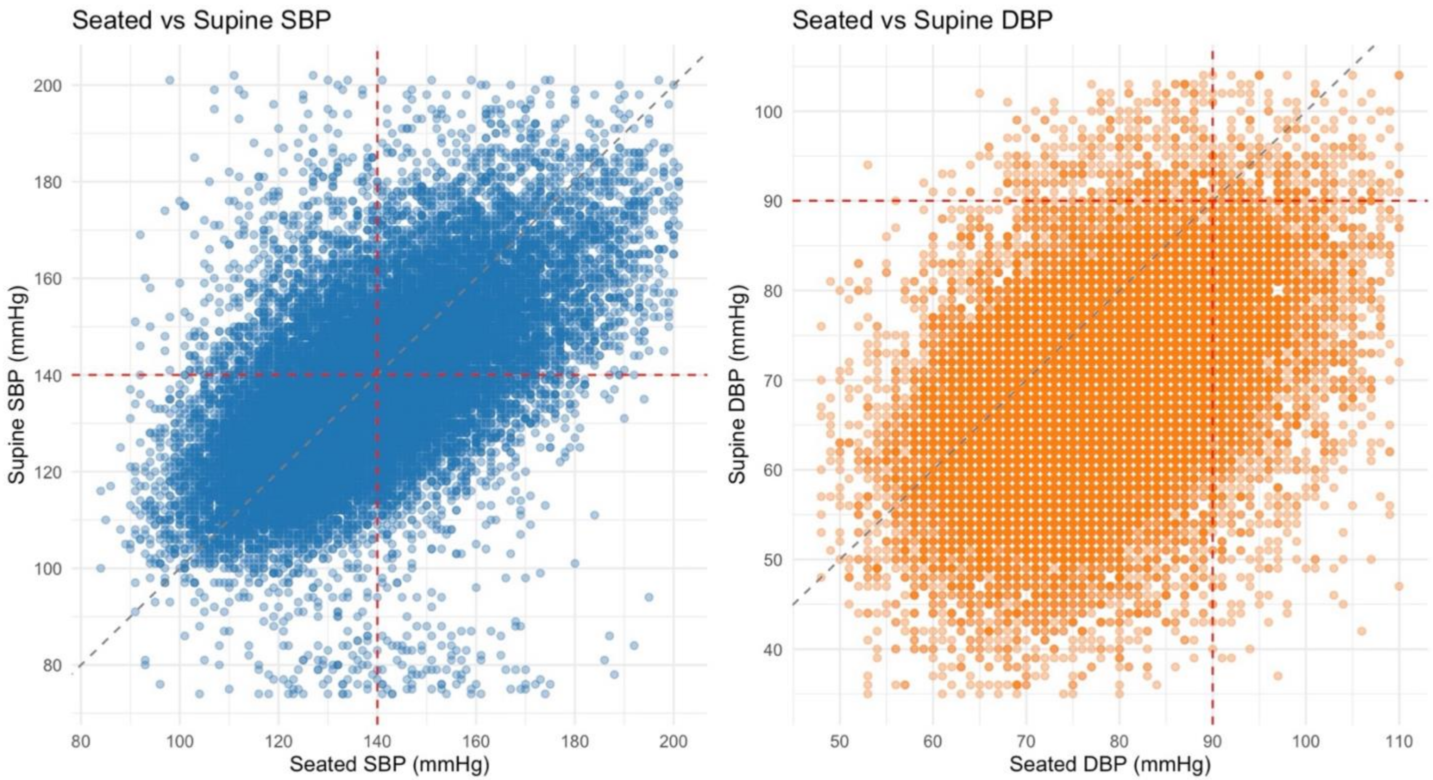

Panel A shows the correlation between seated and supine SBP, and Panel B illustrates the relationship between seated and supine DBP. Red dashed lines indicate the hypertension thresholds (SBP ≥140 mmHg, DBP ≥90 mmHg) for seated and supine positions.

Abbreviation: DBP, diastolic blood pressure; SBP, systolic blood pressure.
